# Indigo Naturalis Suppresses Colonic Oxidative Stress and Th1/Th17 Responses of DSS-Induced Colitis in Mice

**DOI:** 10.1155/2019/9480945

**Published:** 2019-10-13

**Authors:** Hai-tao Xiao, Jiao Peng, Bo Wen, Dong-dong Hu, Xiao-peng Hu, Xiang-chun Shen, Zhi-gang Liu, Zhen-dan He, Zhao-xiang Bian

**Affiliations:** ^1^School of Pharmaceutical Sciences, Guangdong Key Laboratory for Genome Stability & Human Disease Prevention, Shenzhen Key Laboratory of Novel Natural Health Care Products, Innovation Platform for Natural Small Molecule Drugs, Engineering Laboratory of Shenzhen Natural Small Molecule Innovative Drugs, Health Science Center, Shenzhen University, Shenzhen 518060, China; ^2^School of Chinese Medicine, Hong Kong Baptist University, Kowloon, Hong Kong; ^3^The Key Laboratory of Pharmacology and Druggability for Natural Medicines, Department of Education, Guizhou Medical University, Guiyang, Guizhou 550025, China; ^4^Department of Pharmacy, Peking University Shenzhen Hospital, Shenzhen 518036, China; ^5^Shenzhen Research Institute and Continuing Education, Hong Kong Baptist University, Shenzhen 518060, China

## Abstract

Indigo naturalis (also known as Qing-dai, or QD), a traditional Chinese medicine, has been widely used as an anticolitis regimen in the clinical practice of Chinese medicine. However, the precise mechanisms behind its efficacy remain unknown. We investigated the protective effects and associated molecular mechanisms of QD in DSS-induced colitis in mice. We found that QD administration attenuated DSS-induced colon shortening, tissue damage, and the disease activity index during the onset of colitis. Moreover, QD administration significantly suppressed colonic MPO activity and increased the activities of colonic T-SOD, CAT, and GSH-Px, as well the expression of p-AMPK and Nrf-2 in colon tissues of colitic mice. In addition, QD was capable of reducing the colonic Th1 and Th17 cell cytokines, the frequencies of Th1 and Th17 cells, and the phosphorylation of p-STAT1 and p-STAT3 in the mesenteric lymph nodes of colitic mice. An *in vitro* assay showed that QD significantly suppressed the differentiation of Th1 and Th17 cells. These findings suggest that QD has the potential to alleviate experimental colitis by suppressing colonic oxidative stress and restraining colonic Th1/Th17 responses, which are associated with activating AMPK/Nrf-2 signals and inhibiting STAT1/STAT3 signals, respectively. These findings also support QD as an effective regimen in the treatment of IBD.

## 1. Introduction

Inflammatory bowel disease (IBD), such as ulcerative colitis and Crohn's disease, represents a group of lifelong recurrent relapsing-remitting intestinal disorders, which are associated with gastrointestinal symptoms, such as weight loss, diarrhea, abdominal pain, internal bleeding, and extensive colonic mucosal and submucosal damage [1, 2]. IBD patients are being diagnosed worldwide; its in-patient admission rate is stabilizing at around 0.3% in Western countries and is rising rapidly in Asia, in parallel with westernization [3]. For example, the in-patient admission rate of IBD in the Yunnan province of China has risen 19.4-fold from 1998 to 2013 [4]. With no cure, its high hospitalization costs and increasing prevalence place an increased stress on healthcare systems. Current medical therapies for IBD focus mainly on inducing remission and preventing relapse, and aminosalicylates, corticosteroids, thiopurines, and biological agents are commonly recommended as the standard treatments for IBD patients [1]. However, these agents are not very satisfactory and have various side effects that limit their therapeutic benefits, particularly for long-term therapy [5]. For example, long-term treatment of aminosalicylates and corticosteroids could induce depression, growth retardation, osteoporosis, and hypertension [5]; and thiopurines such as azathioprine and 6-mercaptopurine could induce bone marrow suppression, pancreatitis, and hepatic toxicity [6]. Thus, there is still a considerable need to improve the treatment of IBD, and significant research efforts are underway to address this unmet need.

Over the past two decades, the understanding of IBD pathogenesis has progressed rapidly, but it is still not completely understood. However, impaired endogenous enzymatic antioxidants, including superoxide dismutases (SOD), catalases (CAT), glutathione peroxidases (GSH-Px), and glutathione reductases (GRx), and the enhanced production of free radicals, such as reactive oxygen species (ROS) and reactive nitrogen species (RNS), are commonly associated with the development of IBD [7]. Excessive free radical production results in considerable colonic inflammation. Clinically, patients with colitis exhibit an overproduction of ROS, which has a pivotal role in IBD pathogenesis [8]. In addition, the dysregulation of mucosal CD4^+^ T cells also plays a major role in the development of IBD, which impedes the resolution of inflammation and instead results in disease perpetuation and tissue injury [9]. CD4^+^ T cells mainly contain effector CD4^+^ T cells such as T helper 1 (Th1), Th2, Th17, and regulatory T cells (Tregs). Studies have shown that intestinal immune homeostasis depends on the regulation and balance of these CD4^+^ T cell subgroups, and the deregulated overexpansion and activation of Th1 and Th17 cells in relation to Tregs can lead to intestinal inflammation, such as IBD [10–12]. Since Th1 and Th17 cells could secrete large amounts of proinflammatory cytokines such as IFN-*γ*, TNF-*α*, IL-1*β*, and IL-17, resulting in persistent intestinal inflammation [13], medications that can suppress colonic oxidative status and inhibit Th1/Th17 responses would be shown to be clinically effective.

Indigo naturalis (in China, also known as Qing Dai, or QD), a dark-blue powder, is sourced from the leaves and branches of various indigo-producing plants, such as *Baphicacanthus cusia* (Nees) Bremek, *Indigofera tinctoria* L., *Isatis indigotica* Fort, *Polygonum tinctoria* Ait, and *Strobilanthes cusia* (*Nees*) *Kuntze* [14]. In traditional Chinese medicine (TCM), QD has been widely used for treating various infectious and inflammatory diseases, such as enteritis, carbuncles, eczema, and psoriasis [14, 15]. A recent multicenter randomized controlled trial and retrospective observational studies have demonstrated that QD could be effective in inducing mucosal healing in patients with UC and intractable UC [16–18]. In addition, we prescribed a QD-predominant (QDP) herbal medicinal formula to our out-patients with intractable UC who failed to respond to treatment with 5-ASA, prednisolone, or infliximab and achieved a good curative effect [19]. In an animal experiment model, Kawai et al. revealed that QD was effective in ameliorating DSS-induced colonic inflammation, and the mechanism was associated with increasing IL-10-producing CD4^+^ T cells and IL-22-producing CD3^−^ROR*γ*t^+^ cells via aryl hydrocarbon receptor activation [20]. However, it is unclear whether QD can ameliorate intestinal inflammation by targeting colonic oxidative stress and effector Th1/Th17 cells. In the present study, we investigate the therapeutic effects of QD on DSS-induced acute colitis in mice and examine its regulatory mechanisms on colonic oxidative stress and effector Th1/Th17 cells.

## 2. Materials and Methods

### 2.1. Reagents

DSS (molecular weight: 36,000 to 50,000) was purchased from MP Biomedicals (Santa Ana, USA). Dimethyl sulphoxide (DMSO), sulfasalazine (SASP) (purity: 98%), hexadecyltrimethylammonium bromide, hematoxylin, eosin, and *o*-dianisidine dihydrochloride were purchased from Sigma-Aldrich (St. Louis, MO, USA). Fetal bovine serum, L-glutamine, penicillin, streptomycin, and minimum essential medium were purchased from Invitrogen (Carlsbad, CA, USA). CD4^+^CD62L^+^ T Cell Isolation Kits were purchased from Miltenyi Biotec (Bergisch Gladbach, Germany). The fluorescent antibodies CD4, IFN-*γ*, IL-17A, FoxP_3_, and CD3/CD28 were obtained from eBioscience (San Diego, CA, USA) or BD Biosciences (San Jose, CA, USA). Antibodies against p-AMPK, AMPK, Nrf-2, p-STAT1, STAT1, p-STAT3, STAT3, and *β*-actin were supplied from Cell Signaling Technology Inc. (Beverly, MA, USA). Recombinant mouse IL-12, IL-6, and TGF-*β* proteins and antibodies IL-4 and IFN-*γ* were purchased from BioLegend (San Diego, CA, USA). IFN-*γ*, IL-17A/F, TNF-*α*, and IL-1*β* ELISA kits were purchased from eBioscience (San Diego, CA). Total superoxide dismutases (T-SOD), CAT, and GSH-Px colorimetric activity assay kits were purchased from Jiancheng Bioengineering Institute (Nanjing, China). An enhanced chemiluminescence kit was supplied by GE Healthcare Bio-Sciences Corp. (Piscataway, NJ, USA).

### 2.2. Indigo Naturalis

Indigo naturalis (QD) was obtained from the dispensary of the TCM, Clinical Division, School of Chinese Medicine, Hong Kong Baptist University, Hong Kong, which was derived from the herbs of *Strobilanthes cusia* (*Nees*) *Kuntze*. The authentication and quality control of QD were performed based on the requirements of the Chinese Pharmacopoeia [21]. The voucher specimen (voucher number: TCM-0110-Q01) was stored in the Research Laboratory, School of Chinese Medicine, Hong Kong Baptist University, Hong Kong. To identify the components in QD, the sample was weighed exactly, ultrasonically extracted with DMSO, and filtered through a syringe filter for subsequent UPLC-QTO-MS analysis.

### 2.3. UPLC-QTOF-MS Analysis of QD

The components in QD were identified using the UPLC-QTOF-MS method, with a small change in the composition of the mobile phase [22]. The mobile phase for the analysis of QD consisted of (A) 0.1% formic acid in water and (B) 0.1% formic acid in acetonitrile. A linear gradient was optimized as follows (flow rate, 0.40 mL/min): 0–2.5 min, 2–5% B; 2.5–10 min, 5–35% B; 10–20 min, 35–75% B; 20–23 min, 75–100% B; 23–26 min, 100% B; 26–26.1 min, 100–2% B; and 26.1–30 min, 2% B.

### 2.4. Animals

Seven to eight-week-old male C57BL/6 mice weighing 20–24 g were purchased from the Laboratory Animal Services Center, at the Chinese University of Hong Kong. The animals were fed a standard rodent diet with free access to water and were kept in rooms maintained at 22 ± 1°C with a 12 h light/dark cycle following international recommendations. All animal procedures and experiments were carried out according to the “Institutional Guidelines and Animal Ordinance” (Department of Health, Hong Kong Special Administrative Region) and approved by the related ethical regulations of Animal Ethics Committees of Hong Kong Baptist University.

### 2.5. Induction of Colitis and Treatment

Colitis was induced by DSS administration as per our previous description in [23]. Briefly, the colitis was induced by feeding the mice 2% DSS drinking water for five days. After five days of DSS administration, the colitic mice were subjected to a disease activity index (DAI) test and selected for the investigation. The colitic mice were randomly divided into the DSS model group, the SASP-treated group, and the QD-treated groups with two different dosages. In parallel, a vehicle control group was set up to receive drinking water without DSS throughout the experimental period. QD was orally administrated to the mice, with a dosage of 0.5 and 1 g/kg/day for 7 days according to the preliminary experiment. SASP was selected as a reference positive agent, and its dosage was set to 0.20 g/kg/day according to the literature [24, 25]. SASP and QD were freshly suspended in 0.5% CMC-Na solution and orally administered to the mice for seven days. The vehicle-treated control group and DSS model group received the same volume of the dosing vehicle.

### 2.6. Evaluation of Colitis

Body weight, stool consistency, and stool bleeding were recorded daily. The DAI was determined by combining the scores for body weight, stool consistency, and stool bleeding. At the end of the experiment, colonic segments were excised to measure the length from the ileocecal junction to the anal verge. Subsequently, colonic tissues were embedded in paraffin, and 5 mm sections were stained with hematoxylin and eosin (H&E) and scored in a blind fashion by an experienced pathologist. Colonic MPO activity was also measured as described in our previous study [23].

### 2.7. RNA Extraction and Quantitative PCR Analysis

The total mRNA of the colon tissue was extracted using TRIzol® (Invitrogen). The cDNA was synthesized using a TaKaRa RNA PCR Kit (AMV) Ver. 3.0 (TaKaRa Bio Inc., Dalian, China), and quantitative real-time PCR (qPCR) was detected using the ABI 7500 Real-Time PCR System, with a SYBR Green Master Mix (Applied Biosystems, Foster, CA, USA). Relative quantification of mRNA expression was normalized with *β*-actin control and analyzed using the delta-delta Ct (2^-*ΔΔ*^CT) method. Primer sequences (forward/reverse) were listed as follows: mouse *β*-actin, forward 5′-TGT CCA CCT TCC AGC AGA TGT-3′ and reverse 5′-AGC TCA GTA ACA GTC CGC CTA GA-3′; mouse ROR*γ*t, forward 5′-GGA GCT CTG CCA GAA TGA GC-3′ and reverse 5′-CAA GGC TCG AAA CAG CTC CAC-3′; mouse T-bet, forward 5′-AGC AAG GAC GGC GAA TGT T-3′ and reverse 5′-GGG TGG ACA TAT AAG CGG TTC-3′; mouse IL-17A, forward 5′-TCG AGA AGA TGC TGG TGG GT-3′ and reverse 5′-CTC TGT TTA GGC TGC CTG GC-3′; mouse IFN-*γ*, forward 5′-TGA GTA TTG CCA AGT TTG AGG TCA-3′ and reverse 5′-CGG CAA CAG CTG GTG GAC-3′; and mouse T-bet, forward 5′-AGC AAG GAC GGC GAA TGT T-3′ and reverse 5′-GGG TGG ACA TAT AAG CGG TTC-3′.

### 2.8. CD4 T Cell Differentiation

Naïve CD4 T cells were purified using CD4^+^CD62L^+^ T Cell Isolation Kits and diluted to the density of 1 × 10^6^ cells per mL in RPML-1640 medium. 1 × 10^5^ naïve CD4 T cells were seeded per well into a 96-well plate and stimulated with plate-bound anti-CD3e Ab (5 *μ*g/mL), soluble anti-CD28 Ab (2 *μ*g/mL), and different polarized conditions, in the presence of indicated concentrations of QD for 72 h at 37°C. To generate Th1 cells, naïve CD4 T cells were cultured with IL-12 (50 ng/mL) and anti-IL-4 Ab (1 *μ*g/mL). For generating Th17 cells, naïve CD4 T cells were cultured with IL-6 (100 ng/mL), TGF-*β* (2 ng/mL), anti-IFN-*γ* (1 *μ*g/mL), and anti-IL-4 Ab (1 *μ*g/mL).

### 2.9. Intracellular Staining

Mesenteric lymph node cells from DSS-colitis mice were collected after the experiment, and polarized naïve CD4 T cells were first restimulated with phorbol myristate acetate (PMA, 50 ng/mL) and ionomycin (500 ng/mL) in the presence of GolgiPlug (BD Biosciences) for 5 h and then stained with anti-CD4 and IFN-*γ* or IL-17A or FoxP_3_ Ab for 45 min after blocking FcR. Acquisition was performed using FACS, and data analysis was conducted using FlowJo software (Tree Star Inc., Ashland, OR).

### 2.10. ELISA and Immunoblot

Colon tissues and lymphocytes were homogenized or lysed in an ice-cold RIPA lysis buffer (Cell Signaling Technology, Danvers, MA, USA) with a 1% (*v*/*v*) protease and 10% (*v*/*v*) phosphatase inhibitor cocktail (Roche, Basel, Switzerland) and centrifuged at 16,000 g at 4°C for 15 min. The supernatants were collected to determine the concentrations of the cytokines IFN-*γ*, IL-17A/F, TNF-*α*, and IL-1*β*, as well as the oxidative stress markers T-SOD, CAT, and GSH-Px, using commercial ELISA kits according to the manufacturers' instructions. The protein expressions of p-AMPK, AMPK, Nrf-2, p-STAT1, STAT1, p-STAT3, STAT3, and *β*-actin were analyzed using Western blotting as described elsewhere.

### 2.11. Statistical Analysis

The data are presented as mean value ± standard deviation. Statistical significances were evaluated using one-way ANOVA, followed by Duncan's multiple range tests. GraphPad Prism 5.0 software (GraphPad Software Inc., San Diego, CA, USA) was used for all calculations, and *p* < .05 was considered statistically significant.

## 3. Results

### 3.1. Identification of Major Components in QD by UPLC-QTOF-MS

Under the optimized conditions [26], the major constituents of QD were well separated and detected within 30 min under positive ESI-MS ion mode (as shown in supplementary Fig. [Supplementary-material supplementary-material-1]).  Table 1 shows that a comparison of the retention time (tR), mass fragment ions (*m*/*z*), and UV absorption characteristics (lambda max) with those of chemical compounds in the literature tentatively identified 23 peak signal chemical components.

### 3.2. QD Ameliorated the Severity of DSS-Induced Colitis in Mice

**Figure 1:**
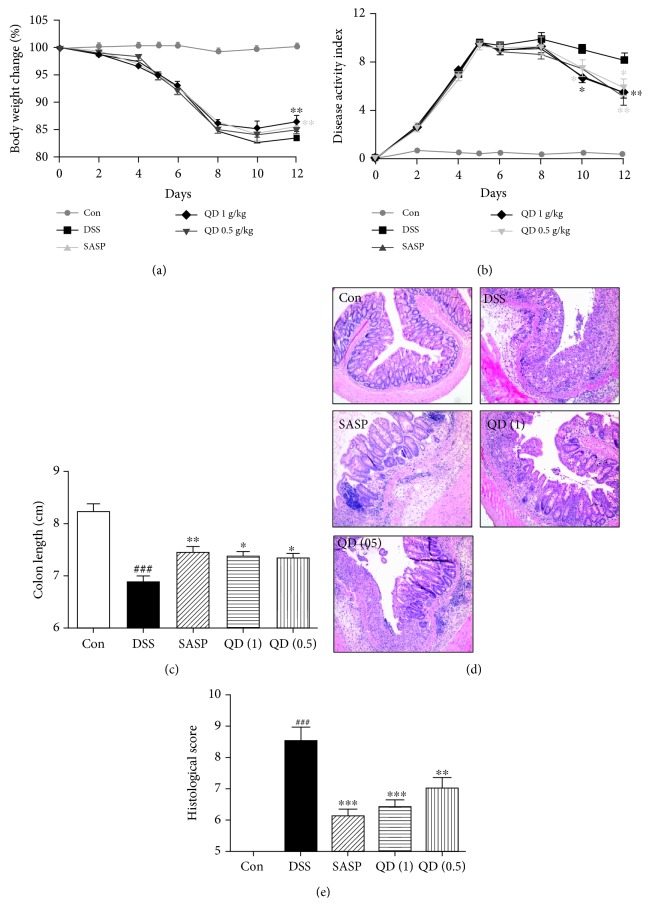
Effects of QD on body loss (a), DAI (b), colon length (c), and histopathological changes (d, e) of mice with DSS-induced colitis. Colitis was induced in all groups except the control group. QD and SASP were administered to mice from days 6 to 12. The change in body weight was taken as the difference between the body weight before the induction of colitis and that immediately before sacrifice on day 13. The DAI was determined by combining the scores for (1) body weight loss, (2) stool consistency, and (3) stool blood. On day 13, the mice were sacrificed, their colon length was measured, and the colonic tissue damage was evaluated by histopathological analysis (H&E staining). Data were expressed as mean ± SD (*n* = 6–8). ^###^*p* < .001, compared with the control group; ^∗^*p* < .05, ^∗∗^*p* < .01, and ^∗∗∗^*p* < .001, compared with the DSS model group.

In the present study, the therapeutic effect of QD was assessed in a murine model of DSS-induced colitis. As shown in Figure 1, oral administration of QD greatly rescued the body loss (Figure 1(a)), as well as significantly reduced DAI (Figure 1(b)), a clinical parameter reflecting the severity of weight loss, rectal bleeding, and stool consistency, compared to the DSS model group (*p* < .05 and *p* < .01, respectively), which even showed a comparative effect with SASP, a first-line drug for UC at a dosage of 0.2 g/kg. In addition, mice with DSS-induced colitis exhibited a shortened colon length when compared to the control mice. As shown in Figure 1(c), QD treatment significantly abated the situation of colon shortening (*p* < .05) and ameliorated histopathological changes such as crypt destruction, epithelial and goblet cell loss, and inflammatory cell infiltration (Figure 1(d)). Compared to the DSS model group, QD-treated groups showed much lower histopathological scores (Figure 1(e)).

### 3.3. QD Suppressed Colonic Oxidative Status DSS-Induced Colitis in Mice

**Figure 2:**
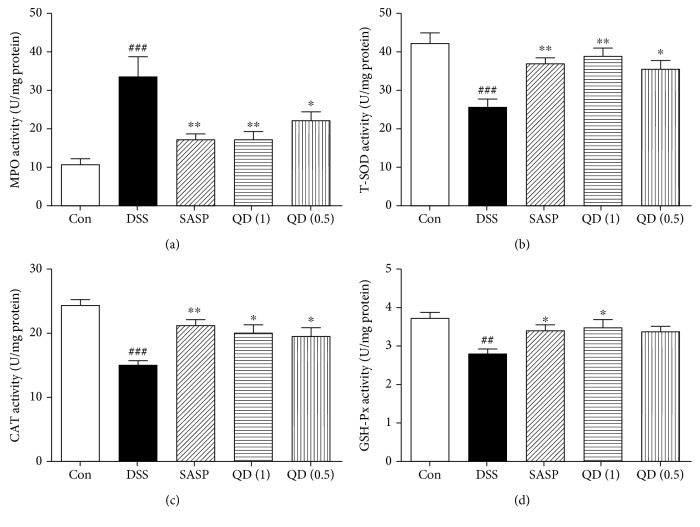
Effects of QD on MPO, T-SOD, GSH-Px, and CAT activities in colon tissues of mice with DSS-induced colitis. Colitis was induced in all groups except the control group. QD and SASP were administered to mice from days 6 to 12. On day 13, the mice were sacrificed and the MPO, T-SOD, GSH-Px, and CAT activities in the colon tissues were measured. Data were expressed as mean ± SD (*n* = 6–8). ^##^*p* < .01 and ^###^*p* < .001, compared with the control group; ^∗^*p* < .05 and ^∗∗^*p* < .01, compared with the DSS model group.

Oxidative stress is one of the critical pathogenic factors for colonic inflammation, and MPO is known as a promoting agent for oxidative stress. Notably, colonic MPO activity was drastically increased in DSS-treated mice compared with the control mice, which was significantly reversed by QD treatment, as shown in Figure 2(a). Accordingly, the activities of antioxidative enzymes including T-SOD, CAT, and GSH-Px in colon tissues were significantly decreased in DSS-treated mice compared with the control mice. Compared with the DSS group, QD at both dosages significantly increased the activities of T-SOD (38.63 ± 5.60 and 35.83 ± 5.60, respectively, vs. 25.14 ± 5.94), CAT (20.09 ± 2.73 and 19.67 ± 2.30, respectively, vs. 14.90 ± 2.40), and GSH-Px (3.50 ± 0.41 and 3.36 ± 0.38, respectively, vs. 2.75 ± 0.45) in the colon of DSS-treated mice (Figures 2(b)–2(d)).

### 3.4. QD Upregulated AMPK and Nrf-2 Activation in the Colon of DSS-Treated Mice

**Figure 3:**
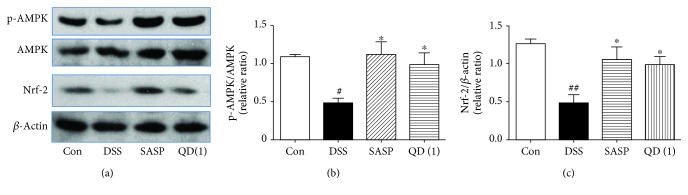
Effects of QD on the expression of p-AMPK, AMPK, and Nrf-2 in colon tissues of mice with DSS-induced colitis. Colitis was induced in all groups except the control group. QD and SASP were administered to mice from days 6 to 12. On day 13, the mice were sacrificed and the expressions of p-AMPK, AMPK, and Nrf-2 in colon tissues were measured using Western blots. Data were expressed as mean ± SD (*n* = 3–4). ^#^*p* < .05 and ^##^*p* < .01, compared with the control group; ^∗^*p* < .05, compared with the DSS model group.

It has been reported that activation of the AMPK/Nrf-2 pathway reduces intracellular ROS levels to prevent cellular oxidative stress damage [27]. We further examined the effects of QD on the expression of p-AMPK, AMPK, and Nrf-2 in the colon of DSS-treated mice. As shown in Figures 3(a)–3(c), colonic p-AMPK and Nrf-2 expressions were drastically decreased in DSS-treated mice compared with the control mice, but QD treatment could significantly reverse those decreases (*p* < .05).

### 3.5. QD Suppressed the Expression of Th1 and Th17 Cell-Related Transcription Factors and Cytokines in the Colon of DSS-Treated Mice

**Figure 4:**
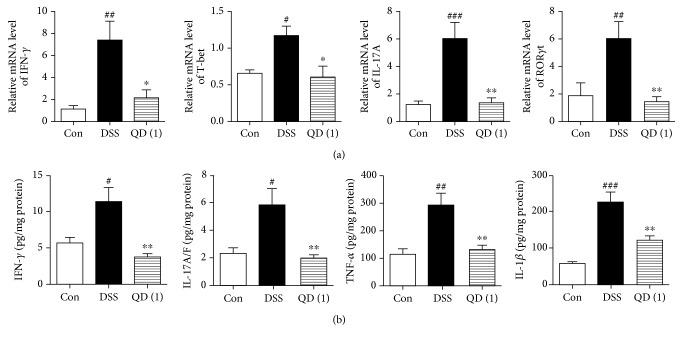
Effects of QD on mRNA and protein expression of Th1/Th17-related cytokines and transcription factors in colon tissues of mice with DSS-induced colitis: (a) mRNA expression of IFN-*γ*, T-bet, IL-17A, and ROR*γ*t; (b) protein expression of IFN-*γ*, IL-17A/F, TNF-*α*, and IL-1*β*. Colitis was induced in all groups except the control group. QD and SASP were administered to mice from days 6 to 12. On day 13, the mice were sacrificed and mRNA expressions of IFN-*γ*, T-bet, IL-17A, and ROR*γ*t in the colon tissues were measured using RT-PCR. Protein expressions of IFN-*γ*, IL-17A/F, TNF-*α*, and IL-1*β* in the colon tissues were measured using ELISA. Data were expressed as mean ± SD (*n* = 6–8). ^#^*p* < .05, ^##^*p* < .01, and ^###^*p* < .001, compared with the control group; ^∗^*p* < .05 and ^∗∗^*p* < .01, compared with the DSS model group.

Mice with DSS-induced acute colitis display a Th1/Th17-characterized cytokine pattern, which shows some similarities to the cytokine profile of human IBD [28]. We therefore examined the effects of QD on Th1 and Th17 cell-related transcription factors and cytokines in the colon of DSS-treated mice. As shown in Figure 4(a), the mRNA expression of IFN-*γ*, IL-17A, T-bet, and ROR*γ*t was remarkably induced by DSS. By contrast, the increased mRNA expression of IFN-*γ*, IL-17A, T-bet, and ROR*γ*t following DSS treatment were significantly decreased after receiving QD treatment. In parallel, the augmented levels of proinflammatory cytokines IFN-*γ*, IL-17A/F, TNF-*α*, and IL-1*β* in the colon of DSS-treated mice were also significantly suppressed by QD treatment (*p* < .01) (as shown in Figure 4(b)).

### 3.6. QD Reduced the Frequencies of Th1 and Th17 Cells in Mesenteric Lymph Nodes of DSS-Treated Mice

**Figure 5:**
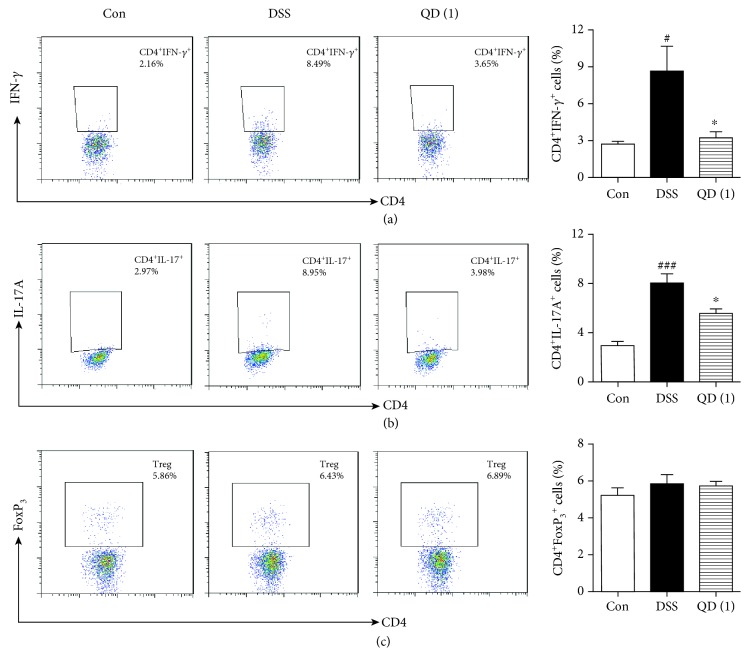
Effects of QD on the frequencies of Th1 (a), Th17 (b), and Treg (c) cells in mesenteric lymph nodes of mice with DSS-induced colitis. Colitis was induced in all groups except the control group. QD was administered to mice from days 6 to 12. On day 13, the mice were sacrificed and the frequencies of Th1, Th17, and Treg cells in mesenteric lymph nodes were measured using flow cytometry. Data were expressed as mean ± SD (*n* = 6–8). ^#^*p* < .05 and ^###^*p* < .001, compared with the control group; ^∗^*p* < .05, compared with the DSS model group.

Since previous results showed that QD affected the expression of Th1 and Th17 cell-related transcription factors and cytokines in the colon of DSS-treated mice, we further examined the effects of QD on the frequencies of Th1 and Th17 cells in mesenteric lymph nodes. As shown in Figure 5, the frequencies of Th1 and Th17 cells in mesenteric lymph nodes were remarkably increased after DSS treatment (*p* < .05 and *p* < .001, respectively). By contrast, the increased Th1 and Th17 cells were greatly decreased after QD administration (*p* < .05) (Figures 5(a) and 5(b)). However, the frequency of CD4^+^FoxP_3_^+^ cells did not show any significant changes after the DSS challenge and QD treatment (Figure 5(c)).

### 3.7. QD Suppressed p-STAT1 and p-STAT3 Phosphorylation in the Mesenteric Lymph Nodes of DSS-Treated Mice

**Figure 6:**
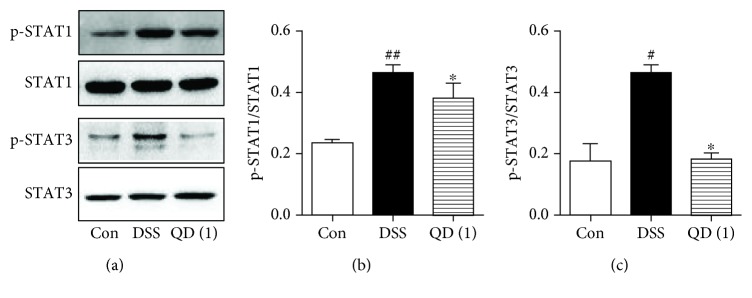
Effects of QD on the phosphorylation of p-STAT1 and p-STAT3 in mesenteric lymph nodes of mice with DSS-induced colitis. Colitis was induced in all groups except the control group. QD was administered to mice from days 6 to 12. On day 13, the mice were sacrificed and the expressions of p-STAT1, STAT1, p-STAT3, and STAT3 in mesenteric lymph nodes were measured using Western blots. Data were expressed as mean ± SD (*n* = 3–4). ^#^*p* < .05 and ^##^*p* < .01, compared with the control group; ^∗^*p* < .05, compared with the DSS model group.

STAT1 and STAT3 are the crucial transcription factors for Th1 and Th17 responses, respectively [28]. We thus examined the effects of QD on the expression of p-STAT1, STAT1, p-STAT3, and STAT3 in the mesenteric lymph nodes of DSS-treated mice. Notably, DSS treatment significantly increased p-STAT1 and p-STAT3 phosphorylation in the mesenteric lymph nodes of mice, and QD administration could significantly inhibit their activation (as shown in Figures 6(a)–6(c)).

### 3.8. QD Suppressed Th1 and Th17 Cell Differentiation *In Vitro*

**Figure 7:**
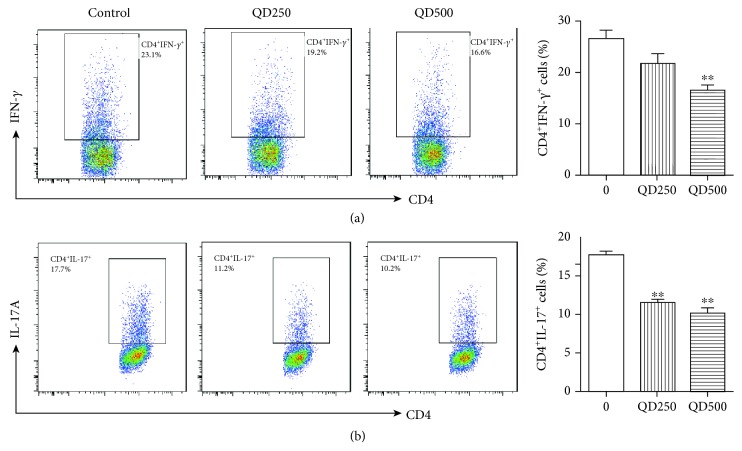
Effects of QD on Th1 (a) and Th17 (b) differentiation in vitro. Naïve CD4 T cells from C57BL/6 mice were incubated under Th1 or Th17 polarizing conditions in the presence or absence of indicated concentrations of QD with anti-CD3/CD28 stimulation for three days. The phenotypes of Th1 and Th17 cells were analyzed using flow cytometry. Data were expressed as mean ± SD (*n* = 3). ^∗∗^*p* < .01, compared with the control group.

To further investigate the protective mechanism of QD against Th1 and Th17 cells, we examined the effects of QD on Th1 and Th17 cell differentiation. The results showed that QD at dosages of 250 and 500 *μ*g/mL could significantly inhibit Th1 and Th17 cells differentiation (Figures 7(a) and 7(b)).

## 4. Discussion

Despite the increased incidences of IBD, there is still no curative therapy for IBD, and current treatments that focus mainly on relieving symptoms often have little beneficial effect or unwanted side effects. When concerning the helplessness of chemical drugs, IBD patients naturally prefer to seek help from traditional herbal medicine (THM), since they believe that the ingredients of THM are all biological, organic, and natural materials that are mostly harmless to humans [29]. QD, as one of most famous heat-clearing and detoxicating THMs, is often prescribed to IBD patients. Although QD is clinically effective, its action mechanisms remain unclear. In the present study, we showed that QD exhibits a potent anticolitis effect in mice with experimental colitis by suppressing colonic oxidative stress and restraining colonic Th1/Th17 responses, which are associated with activating AMPK/Nrf-2 signals and inhibiting STAT1/STAT3 signals, respectively.

It is well known that oxidative stress is a mechanism underlying the pathophysiology of IBD. With the production of excessive ROS, the endogenous self-antioxidant defenses would be overwhelmed, resulting in mucosal disruption and ulceration with infiltration of massive inflammatory cells in the colon tissues [7]. MPO is a marker of tissue injury and neutrophil infiltration, as well as a promoting agent for oxidative stress [30]. Previous studies have well documented that the increase of MPO is closely linked to the severity of IBD [23, 30, 31]. In the present study, we found that colonic MPO activity in DSS-treated mice was drastically increased compared with the control mice, indicating extensive neutrophil infiltration into the colonic tissues. In addition, the infiltration of massive inflammatory cells, such as neutrophils within the mucosa, produced and released a large amount of ROS in an uncontrolled manner. Highly localized concentrations of ROS could attack and inactivate endogenous antioxidant defenses, such as GSH-Px, T-SOD, and CAT, thus preventing the neutralization of ROS [32, 33]. We also observed a decrease in colonic GSH-Px, T-SOD, and CAT activities in DSS-treated mice compared with those in the control mice, which were consistent with previous reports [32, 34]. Notably, administration of QD significantly restrained the elevation of colonic MPO activity and decreased colonic GSH-Px, T-SOD, and CAT activities in DSS-treated mice. Also, previous studies have revealed that QD administration significantly suppressed inflammations by scavenging ROS in UC patients and attenuated the ROS production from the NSAID-induced mitochondrial alteration in the gastrointestinal epithelial cells [16, 35]. Taken together, these findings indicate that QD is effective for inhibiting oxidative stress and boosting antioxidant defenses.

Recent evidence has indicated that AMPK activation plays pivotal roles in regulating epithelial functions in experimental colitis in mice, and activation of the AMPK pathway reduces intracellular ROS levels to prevent cellular oxidative stress damage [27, 36]. Nuclear factor erythroid 2-related factor 2 (Nrf-2) was considered important for protecting against the oxidative stress in cells, which is regulated by AMPK [27, 37]. Previous studies have reported a significant decrease in the mRNA and protein expressions of Nrf-2 and p-AMPK in the colon tissues of various experimental colitis, showing that reversing the decrease of Nrf-2 or p-AMPK ameliorates colitis [27, 36, 38, 39]. Our data demonstrated that DSS treatment significantly decreased the expression of Nrf-2 or p-AMPK in the colon tissues, which can be effectively reversed by QD administration. These results indicate that the antioxidative potential of QD on DSS-induced colitis was attributed to activating the AMPK/Nrf-2 signaling pathway.

Excessive effector T cells such as Th1 and Th17 cells are major players in the disease process, and targeting Th1/Th17 to attenuate the severity of IBD has been well documented [40]. To mimic this disease in mice, DSS-induced colitis is a well-established animal model, which displays a predominant cytokine pattern of a Th1/Th17 type closely resembling those in IBD patients [28]. Using this model, we found that QD could greatly suppress the gene expression of Th1 and Th17 cell cytokines IFN-*γ* and IL-17A and their transcription factors T-bet and ROR*γ*t, as well as the production of Th1/Th17-related cytokines, namely, IFN-*γ*, IL-17A/F, TNF-*α*, and IL-1*β*. Moreover, flow cytometric detection showed that both Th1 (CD4^+^IFN-*γ*^+^) and Th17 (CD4^+^IL-17A^+^) cells in mesenteric lymph nodes of DSS-treated mice and the differentiation of Th1 and Th17 cells *in vitro* were significantly reduced after QD treatment. These results clearly revealed that QD suppresses Th1/Th17 responses in DSS-induced colitis.

STATs are a family of nuclear proteins mediating the actions of numerous cytokines. Among them, STAT1 plays a critical role in the signal transduction pathway of IFN-*γ*, which drives Th1 cell differentiation and activation [41], and STAT3 is a crucial transcription factor for Th17 cell development and in regulating the production of IL-17A [28]. Our data demonstrated that the phosphorylation of p-STAT1 and p-STAT3 in the mesenteric lymph nodes of DSS-induced colitic mice was significantly increased compared with the expression in the control mice. Additionally, QD could greatly suppress activation of both STAT1 and STAT3, indicating that suppressing STAT1 and STAT3 activation could contribute to the inhibitory effects of QD on Th1/Th17 responses.

## 5. Conclusion

The present study demonstrates that QD exhibits a potent therapeutic effect on DSS-induced colitis, which may be associated with suppressing colonic oxidative stress and restraining colonic Th1/Th17 responses, which are associated with activating AMPK/Nrf-2 signaling pathways and inhibiting STAT1/STAT3 signaling pathways, respectively. These findings support that QD is an effective regimen for the treatment of IBD.

## Figures and Tables

**Table 1:** Main components identified in QD.

Peak	tR (min)	Assigned identity	Molecular formula
1	0.976	Butanedioic acid	C_4_H_6_O_4_
2	5.771	Isatin	C_8_H_5_NO_2_
3	5.916	2,3-Dihydro-4-hydroxy-2-oxo-1H-indole-3-acetic acid	C_10_H_8_N_2_O_2_
4	6.497	Deoxyvascinone	C_11_H_10_N_2_O
5	7.717	10H-indolo [3, 2-b] quinoline	C_15_H_10_N_2_
6	8.884	3-(2-Hydroxyphenyl)-4(3H)-quinazolinone	C_14_H_10_N_2_O_2_
7	8.998	10H-indolo[3,2-b]quinoline-11-carboxylic acid amide	C_16_H_11_N_3_O
8	9.597	Tryptanthrin	C_15_H_8_N_2_O_2_
9	11.491	Syringin	C_17_H_24_O_9_
10	11.902	3-(2-Carboxyphenyl)-4(3H)-quinazolinone	C_15_H_10_N_2_O_3_
11	13.250	Indigo	C_16_H_10_N_2_O_2_
12	14.006	Indican	C_14_H_17_NO_6_
13	14.158	Indirubin	C_16_H_10_N_2_O_2_
14	14.640	2-[Cyano(3-indolyl)methylene]-3-indolone	C_18_H_11_N_3_O
15	19.153	Octadecanoic acid	C_18_H_36_O_3_
16	19.332	Bisindigotin	C_32_H_18_N_4_O_2_
17	20.554	Qingdainone	C_23_H_13_N_3_O_2_
18	20.848	Betulin	C_30_H_50_O_2_
19	21.460	Sugiol	C_20_H_28_O_2_
20	22.815	Rotenol	C_23_H_24_O_6_
21	23.433	Sitosterol	C_32_H_53_N_7_O_2_
22	24.699	Daucosterol	C_35_H_60_O_6_
23	26.831	Clerosterol	C_29_H_48_O

## Data Availability

The data used or analyzed during the current study are available from the corresponding authors on reasonable request.

## Supplementary Materials

Supplementary Materials Supplementary Figure 1: total ion chromatograms (TICs) in the positive mode of the QD extract.
