# Supplementary material for: Indigo Naturalis Suppresses Colonic Oxidative Stress and Th1/Th17 Responses of DSS-Induced Colitis in Mice
Source: Oxid Med Cell Longev. 2019 Oct 13;2019:9480945. doi: 10.1155/2019/9480945 (PMC6815543; doi:10.1155/2019/9480945)
Supplement: Supplementary Materials — Supplementary Figure 1: total ion chromatograms (TICs) in the positive mode of the QD extract. [file 9480945.f1.pdf]

Indigo naturalis suppresses colonic oxidative stress and Th1/Th17 responses of DSS-induced colitis in mice

Hai-tao Xiao[1,2,3], Jiao Peng[4], Bo Wen[2,3], Dong-dong Hu[2], Xiao-peng Hu[1], Xiang-chun Shen[3], Zhi-gang Liu[1], Zhen-dan He[1*], Zhao-xiang Bian[2,5*]

[1]School of Pharmaceutical Sciences, Guangdong Key Laboratory for Genome Stability & Human Disease Prevention, Shenzhen Key Laboratory of Novel Natural Health Care Products, Innovation Platform for Natural small molecule Drugs, Engineering Laboratory of Shenzhen Natural small molecule Innovative Drugs, Health Science Center, Shenzhen University, Shenzhen 518060, China

[2]School of Chinese Medicine, Hong Kong Baptist University, Kowloon, Hong Kong

[3]The Key Laboratory of Pharmacology and Druggability for Natural Medicines, Department of Education, Guizhou Medical University, Guiyang, Guizhou 550025, China

[4]Department of Pharmacy, Peking University Shenzhen Hospital, Shenzhen 518036, China

[5]Shenzhen Research Institute and Continuing Education, Hong Kong Baptist University, Shenzhen 518060, China

Correspondence:

Prof. Zhao-xiang Bian, School of Chinese Medicine, Hong Kong Baptist University, Kowloon, Hong Kong. E-mail: bzxiang@hkbu.edu.hk；Prof. Zhen-dan He, School of Pharmaceutical Sciences, Health Science Center, Shenzhen University, Shenzhen 518060, China; E-mail: hezhendan@szu.edu.cn.

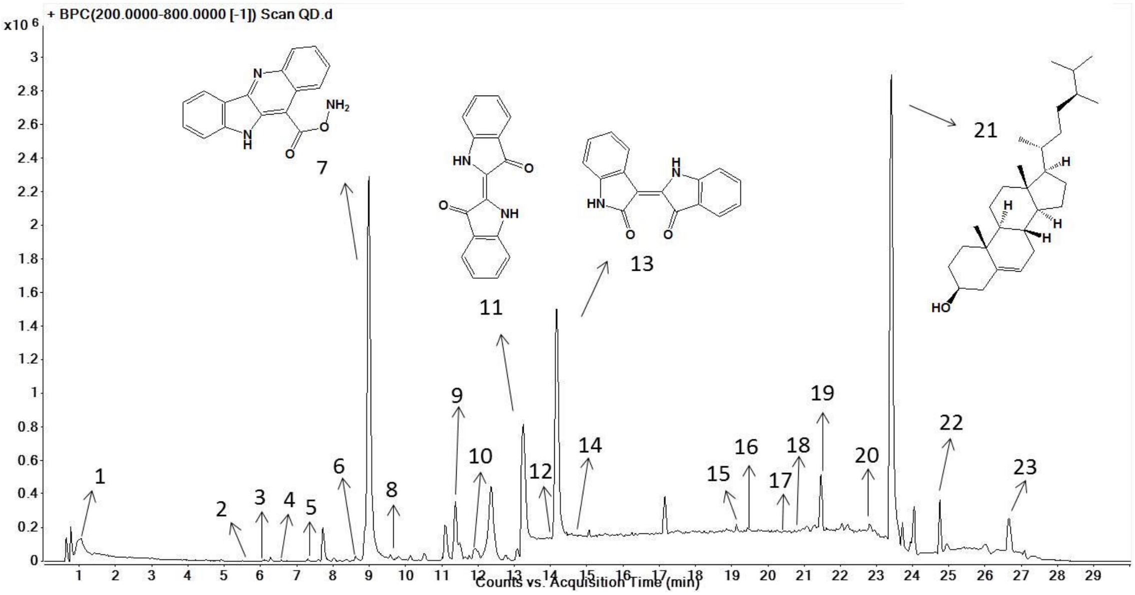

Supplementary Fig. 1. Total ion chromatograms (TICs) in positive mode of QD extract. **7**: 10H-indolo[3,2-b]quinoline-11-carboxylic acid amide; **11**: indigo; **13**: indirubin; and **21**: sitosterol.
